# Confusion in Acute Occipital Infarcts: Why Blame Delirium?

**DOI:** 10.7759/cureus.118289

**Published:** 2026-10-21

**Authors:** Chloe R Plater, Amy Green, Hakim Eluzai

**Affiliations:** 1 Hospital-Based Medicine, University Hospitals Dorset NHS Foundation Trust, Bournemouth, GBR; 2 General Internal Medicine, Dorset County Hospital NHS Foundation Trust, Dorchester, GBR; 3 Stroke Medicine, University Hospitals Dorset NHS Foundation Trust, Bournemouth, GBR

**Keywords:** anton syndrome, confusion, delirium, occipital stroke, visual fields

## Abstract

Acute occipital infarction is a diagnostic challenge due to overlapping symptoms with delirium, another common hospital presentation. We describe a 94-year-old patient presenting with acute confusion and visual changes on imaging. CT imaging shows bilateral occipital infarcts, resulting in a display of Anton syndrome and impacting the patient’s cognition. She was treated for an acute infarct and managed appropriately under the stroke team with the relevant therapy teams.

We explore the challenges in differentiating causes of delirium and offer a diagnostic aid to prompt swifter identification of occipital infarcts in the absence of obvious cause for delirium. This provides an opportunity to encourage efficient diagnosis of occipital infarcts and to improve awareness for identifying key features early.

## Introduction

Acute confusion is a common reason for hospitalisation of older adults. Delirium, characterised by a disturbance in attention and awareness with a fluctuating course, is a frequent cause and may be precipitated by infection, metabolic disturbance, medications, and other acute illnesses [1,2]. Prompt recognition is essential, as delirium is associated with increased morbidity and mortality, increased length of stay, and functional decline [2].

Acute ischaemic stroke should be considered in patients presenting with acute confusion. Unlike anterior circulation infarcts, which typically cause obvious focal neurological deficits, occipital lobe infarctions present more subtly [3]. Visual field defects, cortical blindness, visual agnosia, alexia, and memory impairment may be overlooked, particularly in patients with pre-existing cognitive impairment or reduced insight [4].

Diagnosis of occipital stroke remains challenging because commonly used screening tools, including FAST (Face, Arms, Speech, Time) and the National Institutes of Health Stroke Scale (NIHSS), have limited sensitivity for posterior circulation stroke, contributing to delays in diagnostic imaging and treatment [3,5,6].

We report a case of acute occipital ischaemic stroke presenting predominantly with confusion and subsequently progressing to bilateral occipital infarction with cortical blindness and features of Anton syndrome. Anton syndrome is a rare neurological process where a patient has cortical blindness but can confabulate their surroundings due to a fixed belief of not being visually impaired. It can sometimes present as hallucinations [7]. This case highlights the importance of maintaining a high index of suspicion and performing careful neurological examination, including formal visual field assessment, when evaluating patients with otherwise unexplained acute confusion. Based on the diagnostic challenges illustrated by this case, we propose a simple clinical tool to highlight features that should prompt clinicians to consider occipital stroke in patients presenting with apparent delirium.

## Case presentation

Presenting complaint 

A 94-year-old woman with a medical history of hypertension, chronic kidney disease, bilateral cataracts, and sensorineural hearing loss presented to the emergency department of a district general hospital with a three-week history of worsening confusion. Her next of kin reported that she had become unable to recognise family members. The patient denied any visual disturbance or weakness; however, her family subsequently reported behaviour suggestive of visual hallucinations. She was reportedly seeing family members and attempting to talk to them and interacting when they were not present.

On admission, she was hypertensive, with a blood pressure of 247/100 mmHg, thought to be related to poor adherence to her prescribed antihypertensive medication. Her blood pressure was initially managed with lisinopril and amlodipine, improving to 107/69 mmHg overnight. Her other observations remained stable, with a heart rate of 87 beats/min, respiratory rate of 17 breaths/min, oxygen saturation of 97% on room air, and afebrile. Prior to admission, she lived independently in a flat without a formal care package and mobilised with a walking stick. She was independent in activities of daily living with some help from her next of kin who lived nearby. She had never smoked and did not drink alcohol. She had previously had no cognitive impairment and had a good level of education in childhood.

Initial neurological examination demonstrated preserved power in all limbs without an obvious focal motor deficit. Visual assessment was challenging because of difficulty engaging the patient with the examination. Her initial National Institutes of Health Stroke Score (NIHSS) was 1, measured from 0-42, with the higher readings indicating greater disability [6]. She scored 1 for being disoriented to time and place. She was initially admitted under the acute medical team and later transferred to the stroke unit, where we were involved with her care.

Investigations

A comprehensive investigation for acute confusion was undertaken; full blood count, C-reactive protein, chest radiography, urine and blood cultures, and computed tomography (CT) of the brain. Blood results and chest radiography were unremarkable, and urine culture did not demonstrate evidence of infection. Her haemoglobin (Hb) was 131 g/L (reference range: 115-165 g/L), white cell count (WCC) was 8.6 × 10⁹/L (reference range: 4.0-11.0 × 10⁹/L), C-reactive protein (CRP) was 3.1 mg/L (reference range: less than 5 mg/L), creatinine was 86 µmol/L (reference range: approximately 45-90 µmol/L), and estimated glomerular filtration rate (eGFR) was 50 mL/min/1.73 m² (normal: ≥90 mL/min/1.73 m²). Thyroid-stimulating hormone (TSH) was 1.85 mIU/L (reference range: 0.4-4.0 mIU/L), and free thyroxine (T4) was 16.7 pmol/L (reference range: 12-22 pmol/L). Her adjusted calcium was 2.6 mmol/L (reference range: 2.2-2.6 mmol/L), and sodium was 135 mmol/L (reference range: 135-145 mmol/L), with other electrolytes within their respective reference ranges. Vitamin B12 was 856 ng/L (reference range: approximately 180-900 ng/L), and folate was 6.2 µg/L (reference range: approximately 3.0-20 µg/L). CT brain imaging demonstrated a subacute left posterior cerebral artery (PCA) territory infarct (Figure 1).

**Figure 1:**
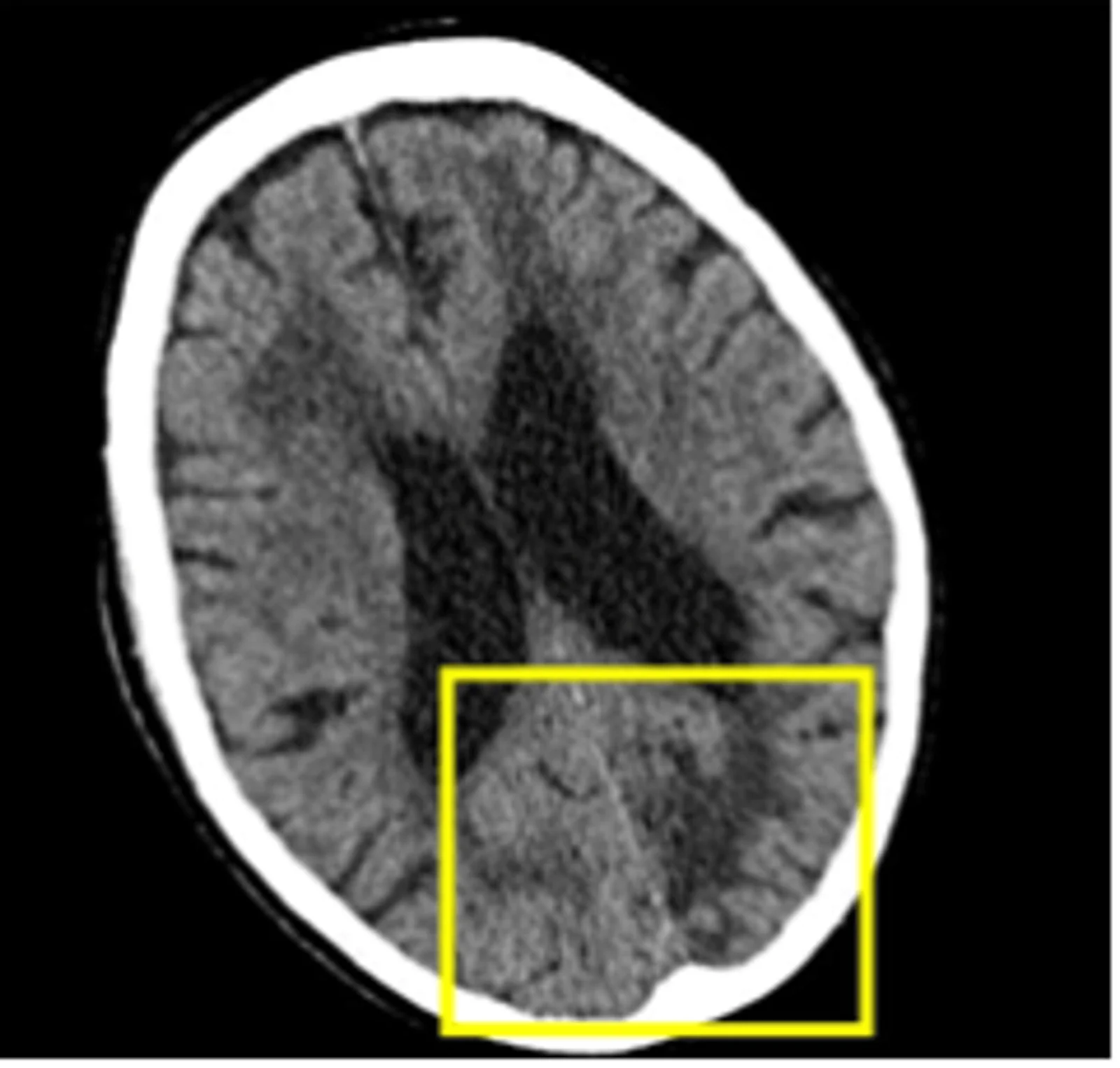
CT head imaging demonstrating localised hypoattenuation with loss of grey-white matter differentiation in the left occipital lobes, suggestive of a subacute or established PCA territory infarct PCA: posterior cerebral artery

At this stage, the differential diagnoses included delirium secondary to stroke, delirium secondary to occult infection, and, given a reported history of weight loss and altered appetite, an intracranial space-occupying lesion.

During the first week of admission, her clinical condition deteriorated. She became increasingly confused and drowsy, with features consistent with hypoactive delirium and further reports of apparent hallucinations. A septic screen was performed, and empirical broad-spectrum intravenous antibiotics (co-trimoxazole 960 mg twice a day) were commenced. At this point, blood results were as follows: WCC was 10.4 × 10⁹/L, CRP was 79 mg/L, Hb was 120 g/L, creatinine was 101 µmol/L, and eGFR was 41 mL/min/1.73 m². Repeat CT brain imaging and subsequent magnetic resonance imaging (MRI) demonstrated new acute bilateral occipital infarcts (Figures 2a, 2b). She received 14 days of aspirin 300 mg. 

**Figure 2:**
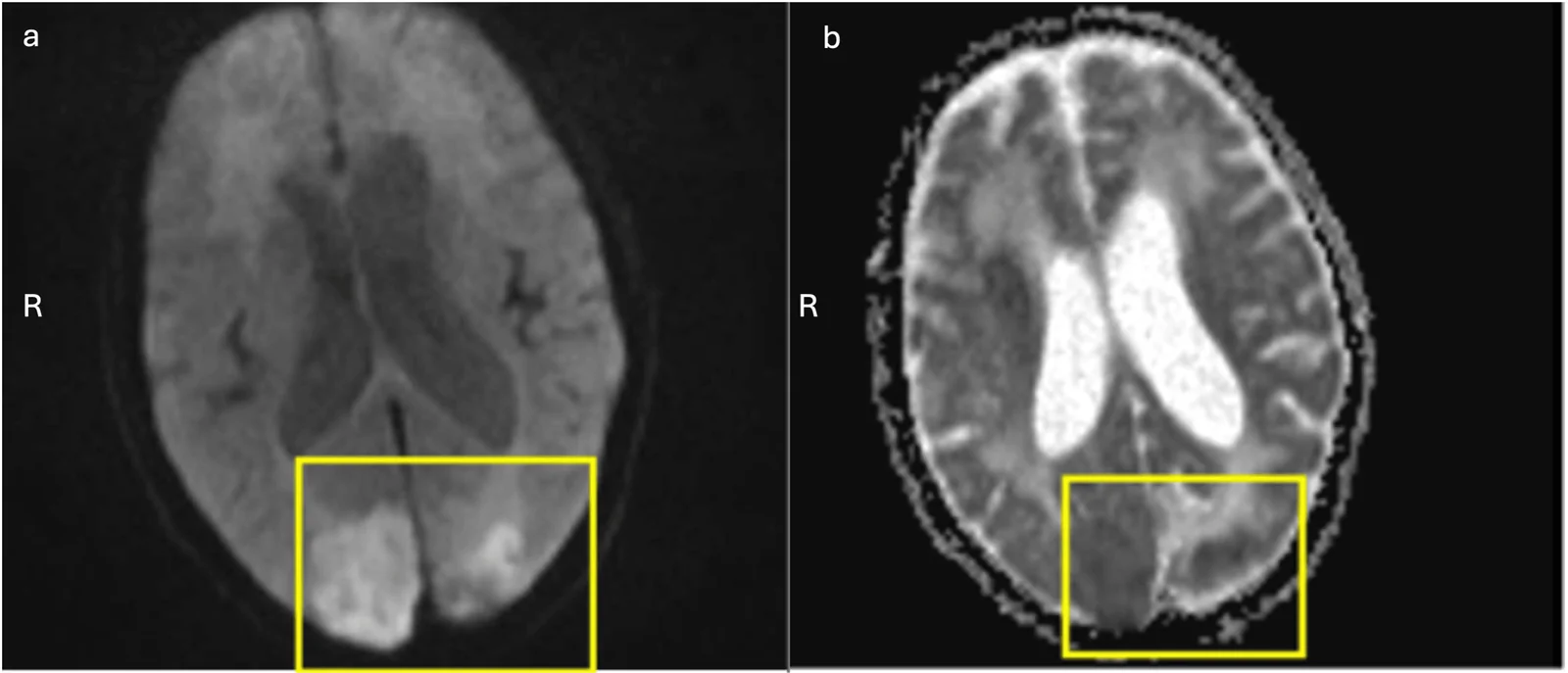
a) Diffusion-weighted MRI and b) Apparent diffusion coefficient (ADC) sequence demonstrating restricted diffusion involving both occipital lobes and the posteromedial parietal lobes consistent with acute bilateral PCA territory infarction The image quality was limited by movement artefacts. PCA: posterior cerebral artery

Management and discharge

The patient was managed on the stroke unit where she was initially loaded on 300mg of aspirin, later switching to single-drug therapy of clopidogrel 75 mg once daily for secondary stroke prevention. She was also given enoxaparin 40 mg once daily for venous thromboembolism prophylaxis. Following further cerebral infarction while receiving clopidogrel, her antiplatelet therapy was changed to ticagrelor 90 mg twice a day for secondary stroke prevention. Coagulation studies were performed and were within the normal range: prothrombin time (PT) was 14.5 seconds (reference range: 11-15 seconds), international normalised ratio (INR) was 1.1 (reference range: 0.8-1.2), and activated partial thromboplastin time (APTT) was 32.6 seconds (reference range: approximately 25-35 seconds). Antibiotics were discontinued prior to the course finishing due to limited evidence of any infection.

Subsequent assessment by the occupational therapy team identified features consistent with cortical blindness. Ward documentation frequently described the patient as ‘hallucinating’; however, formal visual field assessment remained challenging because of difficulty engaging the patient with examination. On observing her within the ward, she would be feeling around her table, trying to locate objects; however, when asked, she would deny not being able to see and would confabulate objects or people around the room. Referrals were made to orthoptics and ophthalmology. Her cortical blindness, apparent visual hallucinations or confabulation, and lack of awareness of her visual impairment were considered consistent with Anton syndrome. 

Cognitive assessment using the Montreal Cognitive Assessment (MoCA) produced a score of 0/30 [8]. The assessment was repeated prior to discharge with the patient again scoring 0/30, indicating persistent severe cognitive impairment following her bilateral occipital infarcts. Interpretation of these scores was limited by her profound visual impairment and difficulties engaging with cognitive impairment.

She was discharged to a nursing home after a period of inpatient therapy. She is booked for an outpatient echocardiogram and will continue lifelong stroke prevention.

## Discussion

The case highlights the diagnostic challenge associated with occipital ischaemic stroke in an older patient presenting predominantly with confusion. Although the initial presentation was compatible with delirium, neuroimaging demonstrated an occipital infarct, with subsequent clinical deterioration corresponding to the development of acute bilateral PCA territory infarction. Delirium and stroke are not mutually exclusive diagnoses, and post-stroke delirium is well-recognised [9]. However, attributing unexplained confusion solely to delirium may lead to delay in recognition of neurological pathology, particularly when typical focal deficits are absent.

Stroke presentation varies according to the vascular territory and brain regions affected. Commonly used screening tools, such as FAST, recognise facial weakness, limb weakness, and speech disturbance but may be less sensitive to posterior circulation presentations. To highlight clinical features that may point to occipital pathology rather than delirium, we propose the mnemonic VISUAL: Visual field abnormality, Impaired insight, Sudden onset or sudden deterioration, Unexplained confusion, Agnosia/alexia/apparent hallucinations, and Low NIHSS does not exclude stroke. VISUAL is intended as an aide-memoire rather than a validated diagnostic tool.

Visual field abnormality is a prominent feature of occipital infarction as visual pathways are frequently affected in occipital stroke, with homonymous hemianopia representing an important clinical finding. Careful assessment of visual fields should therefore be an important component of the neurological exam when posterior circulation stroke is suspected [10]. However, reliable visual field assessment may be difficult in patients with confusion, cognitive impairment, communication difficulties or poor engagement with examination, as demonstrated in this case. Difficulty completing formal testing should therefore not be interpreted as evidence of preserved vision, and observation of visual behaviour may provide additional evidence of visual dysfunction.

Impaired insight and agnosia, alexia, or apparent hallucinations are closely related red flags. This patient’s cortical blindness was not fully recognised due to her lack of awareness of her visual impairment. Her inability to recognise family members and behaviour described as visual hallucinations were initially interpreted as manifestations of acute delirium. Following bilateral occipital infarction and cortical blindness, her lack of insight into her visual deficit and apparent confabulation were considered consistent with Anton syndrome [7]. Anton syndrome is a rare form of visual anosognosia in which patients with cortical blindness deny their visual impairment and may confabulate descriptions of their surroundings [11-13]. These behaviours may be misinterpreted as hallucinations or confusion, particularly in older patients in whom delirium is already considered likely. The discrepancy between impaired vision and the patient’s apparent confidence in their vision should prompt consideration of visual anosognosia.

Sudden onset or sudden deterioration and unexplained confusion emphasise the importance of reassessment when the clinical course is not adequately explained by delirium. In this case, visual and perceptual abnormalities may have been present for three weeks prior to admission, while initial investigation identified no convincing infective or metabolic cause for her confusion. Subsequently, worsening confusion, drowsiness and apparent hallucinations prompted investigation and empirical treatment for infection. However, repeat neuroimaging demonstrated new bilateral occipital infarction. Although delirium may complicate stroke, new or progressive cognitive, visual or perceptual abnormalities should prompt repeat neurological examination and consideration of evolving neurological pathology.

The final component of VISUAL emphasises that a low NIHSS does not exclude stroke. Despite radiological evidence of a left PCA territory infarct, the patient’s initial NIHSS score was only 1. The NIHSS may underestimate the clinical severity of posterior circulation stroke, particularly when visual, perceptual, or cognitive abnormalities predominate. A low NIHSS score should therefore not exclude clinically significant posterior circulation infarction when the history or examination raises suspicion [3,5,14].

VISUAL (described in Table 1) is proposed as a practical aide-memoire of clinical red flags, which should prompt consideration of occipital stroke in patients presenting with apparent delirium. Its individual components are non-specific, and delirium, visual impairment, and stroke can coexist. It should therefore not be used to distinguish stroke from delirium or to determine the need for neuroimaging but rather aim to encourage recognition of otherwise overlooked neurological features of posterior circulation stroke in patients presenting with acute confusion. Further evaluation would be required to determine whether this framework improves recognition of occipital stroke beyond established delirium and stroke assessments.

**Table 1:** Proposed VISUAL aide-memoire for occipital stroke in patients presenting with acute confusion This is a proposed educational red-flag tool, rather than a validated diagnostic or screening test.

VISUAL	Red Flag	Clinical Implication
V	Visual Fields	Assess visual fields and behaviour
I	Impaired insight	Consider visual anosognosia/Anton syndrome
S	Sudden onset or deterioration	Consider acute neurological pathology
U	Unexplained confusion	Reconsider neurological causes if delirium screen is unrevealing
A	Agnosia, alexia or apparent hallucinations	Consider occipital cortical dysfunction
L	Low NIHSS does not exclude stroke	Maintain clinical suspicion in the absence of high NIHSS or positive FAST

## Conclusions

Occipital infarction may present with predominantly cognitive, perceptual, and visual symptoms rather than focal neurological deficits typically associated with acute stroke. In older patients presenting with unexplained confusion, stroke should therefore remain an important differential diagnosis even when FAST is negative or NIHSS is low. There is time wasted concentrating on delirium instead of identifying and treating occipital infarcts.

This case demonstrates how cortical blindness, visual anosognosia, and confabulation associated with Anton syndrome may be misinterpreted as delirium or visual hallucinations. We propose VISUAL as a simple aide-memoire for clinical features that should prompt consideration of occipital stroke in those presenting with apparent delirium. VISUAL is not intended to replace established delirium or stroke assessments and requires evaluation before being used as a validated clinical tool. Instead, it acts as a reminder of the importance of careful visual fields assessment and keeping neurological causes in the differential when the clinical presentation is not fully explained by delirium.
